# Celiac and Non-Celiac Forms of Gluten Sensitivity: Shifting Paradigms of an Old Disease

**DOI:** 10.9734/BMRJ/2013/6083

**Published:** 2013-10

**Authors:** Karol Sestak, Ilana Fortgang

**Affiliations:** 1 Division of Microbiology, Tulane National Primate Research Center and Tulane University School of Medicine, 18703 Three Rivers Road, Covington, LA 70433, USA; 2 Department of Pediatrics, Section of Gastroenterology, Hepatology and Nutrition, Tulane Hospital for Children, 1415 Tulane Avenue, New Orleans, LA 70112, USA

**Keywords:** Celiac, gluten, autoimmunity, tissue transglutaminase

## Abstract

Gluten sensitivity is one of the prominent features of celiac disease (CD) which is an autoimmune disorder characterized by damaged lining of the small intestine. CD was known already to ancient Greeks as κοιλιακός (*keeleeakoss*) i.e. disease of the abdominal cavity hence celiac. Focus of this Commentary article is on rather complex definition of CD and its emerging new forms the example of which is non-celiac gluten sensitivity. It is becoming evident that to formulate more effective treatments, these associations and newly identified disease entities deserve attention from both academic and clinical communities.

## 1. INTRODUCTION

Modern medicine described CD as autoimmune disorder with genetic predisposition while its paradigms are still shifting - thanks to advances brought upon by contemporary molecular techniques. CD-related but understudied and emerging new forms of gluten sensitivity became in recent years subject of continuous debate. While it is estimated that there are at least three million people in the United States alone affected by CD, only a small fraction have been diagnosed [1]. Overall population prevalence ranges between 0.5–1% whereas certain predisposition groups show higher rates. Although CD was once thought to affect predominantly children in Western countries, recent epidemiological data indicate that geographical distribution of this disease is rather associated with consumption of cereal grains and genetic background than with race or age [2]. The pathobiology of classical CD is linked with an autoimmune reaction of genetically predisposed individuals against intestinal tissue transglutaminase (TG2) in the small intestine. This reaction is initially triggered by dietary gluten, since TG2 plays an important role in post-translational modification of glutamine e.g. gliadindeamidation [3]. Diagnosis is based on clinical symptoms (mostly chronic diarrhea and weight loss), histopathological evaluation of small bowel biopsies with expected variable degree of villous atrophy and lamina propria lymphocytic infiltration, as well as serological detection of anti-gliadin, TG2 and/or anti-endomysial IgA antibodies. Treatment requires exclusion of wheat and other cereal grains from the diet. Patients on gluten-free diet respond promptly in most of the cases with resolution of clinical symptoms however, histological changes take longer time to resolve. Some patients develop refractory form of CD and do not respond to dietary gluten withdrawal. These patients require additional treatment with anti-inflammatory drugs. Strict adherence to gluten-free diet is extremely difficult to accomplish due to ubiquity of gluten in general food items. Therefore, an alternative treatment approaches are needed.

## 2. EMERGING EXTRAINTESTINAL AND NON-CELIAC FORMS OF GLUTEN SENSITIVITY

**Fig. 1:**
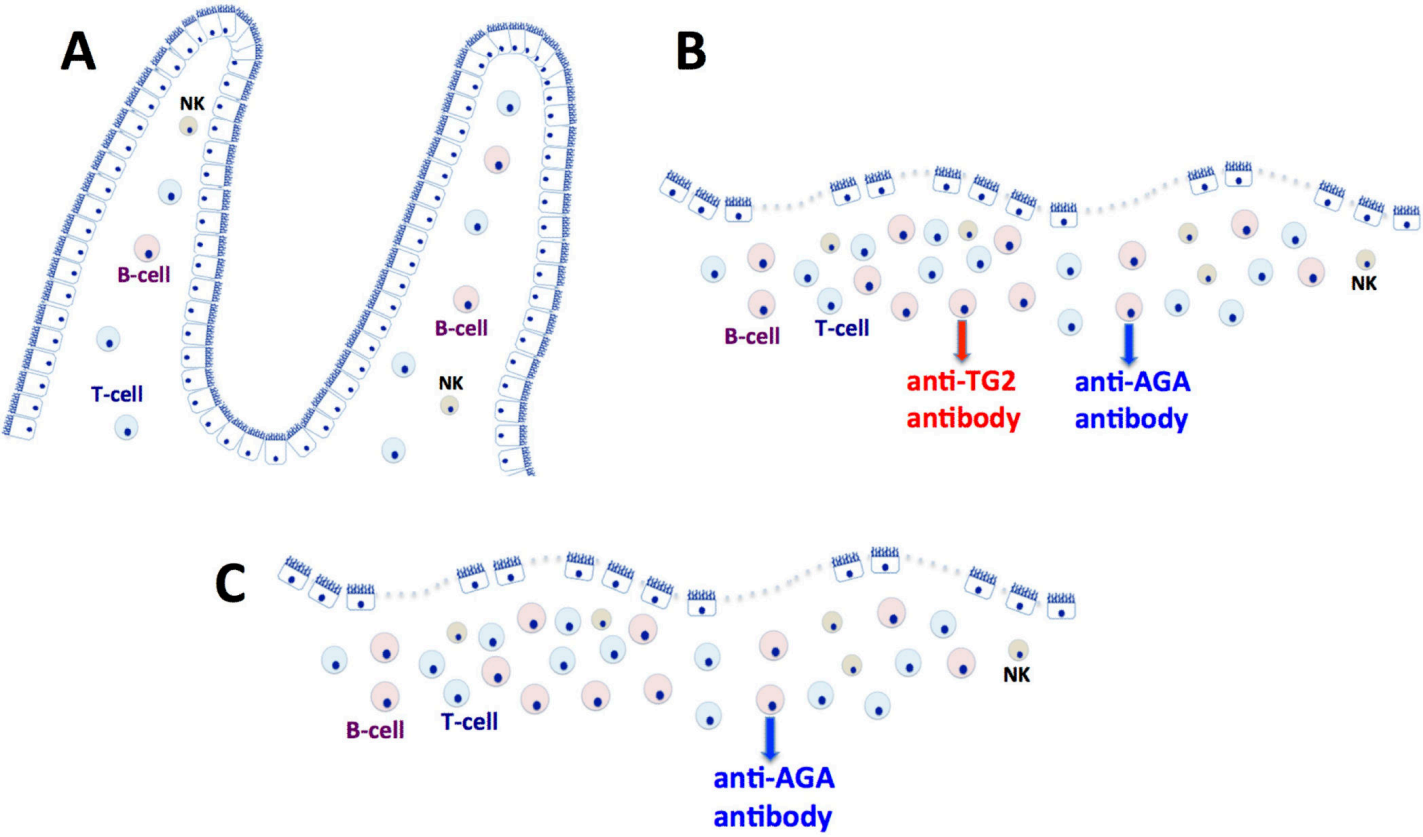
Dietary gluten-induced antibody production Villi of the healthy small intestine consist of epithelial and sub-epithelial layers comprised of several lymphoid cell populations that can either tolerate or recognize foreign antigens (1. A). In celiac patients, dietary gluten triggers production of anti-TG2 and anti-gliadin (AGA) antibodies that can be detected locally in the form of antibody deposits but also systemically in the form of serum antibodies (1. B). Both celiac and non-celiac gluten sensitive (NCGS) patients exhibit leaky gut characterized by compromised epithelial barrier, chronic inflammation of intestinal lamina propria, villous atrophy and increased epithelial permeability. In NCGS, only the AGA but not anti-TG2 antibodies are produced (1. C). Triggering factors and pathogenesis of NGS are not well understood.

The autoimmune etiology of CD is well recognized as the sensitivity to dietary gluten that is strongly associated with *DQ2* (90% of CD patients) and *DQ8* major histocompatibility class II alleles [4]. TG2 occupies important role in pathogenesis of CD due to its function to posttranslationally modify (deamidate) gliadin peptides, resulting in their increased affinity for *DQ2* and *DQ8* alleles. Such reaction leads to CD4+ T cell-mediated inflammatory responses in the small intestine [5–7]. In addition, isoforms of TG2 are also present in other organs such as skin, heart, kidney, brain, bones and uterus. Correspondingly, the extraintestinal (secondary) forms of CD, the examples of which are dermatitis herpetiformis and gluten neuropathy, have been diagnosed [8–9]. In addition to classic CD, wheat allergy has long been recognized and represents as much as 25% of gluten sensitivity overall [10]. Because wheat allergy is mediated by IgE, the pathogenesis of CD and wheat allergy are considered unrelated, although the optimal treatments are the same. More intriguing, however, is another TG2-independent form of gluten sensitivity that is emerging as a discrete new clinical entity [11–12]. Non-celiac gluten sensitivity (NCGS) is more prevalent than CD [13–15], and its pathogenesis is far less understood. For example, celiac *DQ2*/*DQ8* predisposition alleles are still present in greater proportion in NCGS patients (50%) than they are in general population while virtually all CD patients are *DQ2/DQ8* positive [15]. A prospective study showed that of 94 patients with generalized abdominal complaints linked by history to gluten ingestion, 63% had no evidence of CD or wheat allergy, yet all reported relief upon introduction of gluten-free diet [13]. The biological mechanism that would explain such outcome is currently not known. Both CD and NCGS patients develop “leaky guts,” characterized by compromised epithelial barrier, chronic inflammation of intestinal lamina propria, villous atrophy and increased epithelial permeability. However, NCGS patients develop only antibodies to gliadin but not to TG2 (Fig. 1), implying distinct pathogenesis.

## 3. QUESTIONS AND CHALLENGES

Recent surveys indicate that up to one third of irritable bowel syndrome patients are also exhibiting symptoms of NCGS and improve clinically after being placed on gluten-free diet [11]. Despite that epidemiology of NCGS was not yet thoroughly established, it was estimated that NCGS may affect up to ~6% of the general population – a prevalence approximately 6 times higher than that of CD [12]. Still, mechanisms of NCGS pathogenesis, diagnostic markers and predisposition factors are much less understood than those of CD. For example, it is unclear what features of irritable bowel syndrome, if any, enable its synergy with NCGS and/or CD. Some clinical reports have suggested that intestinal dysbiosis might accelerate CD through certain species of rod shaped bacteria with capability to colonize human small intestine. It was proposed that composition of these bacterial species and their impact on gut microbiota is responsible for either increased or decreased inflammatory responses to dietary gluten [16]. Furthermore, these studies suggest that the innate immune responses that develop in these dysbiotic patients reflect the severity of gluten sensitive enteropathy and further derange intestinal homeostasis. In support of this model, recent studies with gluten sensitive rhesus macaques have linked the onset of gluten sensitive enteropathy to decreased production of the interleukin-17/22 [17]. It was hypothesized that this association reflects a compensatory mechanism to preserve the healthy microbiome by delaying the displacement of normal intestinal microflora with pathogenic bacteria. Taken together, NCGS and dysbiosis are amongst those areas of disease that require further studies.

## 4. CONCLUSION

Research that will identify diagnostic markers of NCGS and enable to distinguish it from CD, wheat allergy as well as IBS is called for. To delineate efficient prevention and treatment strategies requires time-scale studies, gluten-modified diets and samples that closely mimic mucosal and systemic involvement of these disease entities. Although clinical research with direct enrollment of patients represents a critical part of this process, ethical constraints render it unfeasible to rely solely on clinical resources. Recently-developed mouse and primate models of CD, that allow synchronized administration of gluten-modified diets as well as multiple time point sample collections offer novel alternatives. In summary, an accurate characterization of newly identified non-celiac forms of gluten sensitivity will require concerted efforts that will lead to better understanding of this disease complex and to formulation of effective prevention and treatment - currently all absent.

## ABBREVIATIONS

CD: Celiac Disease
TG2: Intestinal Tissue Transglutaminase
DQ2 and DQ8: Human Major Histocompatibility Class II Alleles
NCGS: Non-Celiac Gluten Sensitivity
IBS: Inflammatory Bowel Syndrome
AGA: Anti-Gliadin Antibodies
IgA: Immunoglobulin A
IgE: Immunoglobulin E
